# Supplementary material for: On chaotic dynamics in transcription factors and the associated effects in differential gene regulation
Source: Nat Commun. 2019 Jan 8;10:71. doi: 10.1038/s41467-018-07932-1 (PMC6325146; doi:10.1038/s41467-018-07932-1)
Supplement: Supplementary file 1 — Supplementary Information [file 41467_2018_7932_MOESM1_ESM.pdf]

Supplementary Information

Heltberg et. al

Supplementary Note 1

Mathematical Model of NF-κB Network

The network of the transcription factor NF-κB is very important for the mammalian protein production, and several models have therefore been constructed to capture the essential dynamics.
In the model, we consider the NF-κB inside the nucleus (N_n), acting as a transcription factor for many proteins, including I-κB. Here, N_n is the nuclear NF-κB concentration, I_m is the IkB mRNA level, and I is the concentration of cytoplasmic I-κB protein. In the equation for $\dot{N_n}$, the first term models the import of NF-κB into the nucleus, which is inhibited by NF-κB-IκB complexes formed in the cytoplasm. The second term models the formation of these complexes in the nucleus followed by their export into the cytoplasm. The equation for I_{RNA} describes the NF-κB activated transcription of IκB m_{RNA} and the spontaneous degradation of the m_{RNA} with a half-life of $ln(2)/\gamma_m$. The first term in the equation for IκB models translation of IκB m_{RNA} into IκB protein in the cytoplasm, and the second term models the TNF-triggered degradation of IκB in the cytoplasm when it is bound to NF-κB. The triggering stimulus TNF, acts by changing the level of active IκB kinase, $[IKK_a]$, which phosphorylates IκB, resulting eventually in its degradation. This degradation rate is set by the parameter α in the model. It is thus only this protein complex with IKK that can phosphorylate the NF-κB - I-κB complex and make NF-κB active again. This model assumes that there is a constant amount of IKK (IKK_{tot}), which can be in three states: active (IKK_a), inactive(IKK_i) and neutral ($IKK_{tot} - IKK_a - IKK_i$). TNF increases the rate at which neutral IKK is made active, and decreases the rate at which inactive IKK is made neutral. All the parameters used in the NF-κB model are found in the Supplementary Table I.

Parameter in paper	Default value
k_{Nin}	5.4 min^{-1}
k_{Iin}	0.018 min^{-1}
k_t	1.03 $(\mu\text{M})^{-1}.\text{min}^{-1}$
k_{tl}	0.24 min^{-1}
K_I	0.035 μM
K_N	0.029 μM
γ_m	0.017 min^{-1}
α	1.05 $(\mu\text{M})^{-1}.\text{min}^{-1}$
N_{tot}	1. μM
k_a	0.24 min^{-1}
k_i	0.18 min^{-1}
k_p	0.036 min^{-1}
k_{A20}	0.0018 μM
$[IKK]_{tot}$	2.0 μM
$[A20]$	0.0026 μM

Supplementary Table I: Default values of parameters in the model. The first 9 are from Ref. [1] and the next 4 from Ref. [2]. $[IKK]_{tot}$ and $[A20]$ were chosen in order to obtain sustained spiky oscillations with frequency in the range 0.3–1 hr^{-1} when $[TNF]$ is kept fixed at 0.5 (the actual frequency obtained with these values is $\nu_0 = 1/1.8\ hr^{-1}$.)

With these assumptions at hand, we formulate a system with the following five coupled differential equations:

$$\dot{N_n} = k_{Nin}(N_{tot} - N_n)\frac{K_I}{K_I + I} - k_{Iin}I\frac{N_n}{K_N + N_n} \tag{1}$$

$$\dot{I_{RNA}} = k_t N_n^2 - \gamma_m I_{RNA} \tag{2}$$

$$\dot{I} = k_{tl}I_{RNA} - \alpha IKK_a(N_{tot} - N_n)\frac{I}{K_I + I} \tag{3}$$

$$\dot{IKK_a} = k_a \cdot TNF \cdot IKK_n - k_i IKK_a \tag{4}$$

$$\dot{IKK_i} = k_i IKK_a - k_p IKK_i \frac{k_{A20}}{k_{A20} + [A20] \cdot TNF} \tag{5}$$

$$IKK_n = [IKK]_{tot} - IKK_a - IKK_i \tag{6}$$

$$TNF = 0.5 + A\sin(\frac{2\pi}{T}t) \tag{7}$$

A schematic version of this network is shown in Figure 1A, and a more complete discussion is presented in [3]. To describe the impact of dynamically varying concentrations of a transcription factor, we turn to the description of production from genes that are influenced by this. We describe the protein production of a protein P_i with the equations:

$$\dot{m_i} = \gamma_i \frac{N^{h_i}}{N^{h_i} + K_i^{h_i}} - \delta_i m_i, \tag{8}$$

$$\dot{P_i} = \Gamma_i m_i - \Delta_i P_i. \tag{9}$$

Here, the m_i represents the mRNA of gene i, and P_i represents the protein level of gene i. All parameter values in the downstream model can be found in Supplementary Table II:

Parameter in paper	Default value
γ_i	1.0 min^{-1}
$\mathbf{h}_i$	[2-4]
$\mathbf{K}_i$	[1.0-4.5] μM
δ_i	0.03 min^{-1}
Γ_i	1.0 min^{-1}
Δ_i	0.001 min^{-1}

Supplementary Table II: Default values of parameters in the model used for downstream genes.

This means that the transcription factors will enhance the mRNA production through sigmoid shaped curves as seen in Fig. 1E, which we term stimulation profiles. We stress that in the sections to follow, the Hill functions are not important in themselves, and the results are valid for genes that have different sigmoidal shaped stimulation profiles.

Supplementary Note 2

Implementation of Gillespie algorithm

In the Gillespie algorithm we consider a volume V, with a spatially uniform mixture of N chemical species that can react through M different reactions, $R_1 ... R_M$. The number of each of the species is denoted $X_1 ... X_N$. At $t = 0$, we thus consider the initial number of molecules and calculates all reactions. The first goal is now to calculate the PDF, for the time until the next reaction occur
We evaluate the probability that the next reaction is of type ϵ, and it occurs in the time-interval $[t+\tau, t+\tau+dt]$. We therefore consider:

$$P(\tau,\epsilon)d\tau = \overbrace{P_{not}(\tau)}^{\text{No reaction in } [t,t+\tau]} \cdot \overbrace{R_\epsilon d\tau}^{\text{Reaction } \epsilon \text{ occurs}} \tag{10}$$

Therefore we want to describe $P_{not}(\tau)$ in terms of the rates. For an infinitesimal time step the probability for no reaction to appear is:

$$P_{not}(dt) = 1 - \sum_{i=1}^{N} R_i dt \tag{11}$$

We can thus define $\tau \equiv n \cdot dt$ and then:

$$P_{not}(\tau) = P_{not}(dt)^n = \left(1 - \sum_{i=1}^{N} R_i \frac{\tau}{n}\right)^n = e^{-r\tau} \tag{12}$$

$$\text{where} \quad r \equiv \sum_{i=1}^{N} R_i \tag{13}$$

This means that we should generate a random number according to the exponential distribution, and a random number according to a uniform distribution. Here one can use the transformation method, and we can then create the update process, where at each step we jump a step in time τ to next reaction, and picks the reaction according to r. Schematically the Gillespie algorithm can be described as:

- Pick two random numbers, ν_1 and ν_2. Calculate time until next reaction:

$$\tau = -\frac{ln(\nu_1)}{r} \tag{14}$$

  Pick the next reaction:

$$\epsilon = \frac{\sum_{i=1}^{k-1} r_i}{\sum_{j=1}^{n} r_j} < \nu_2 \leq \frac{\sum_{i=1}^{k} r_i}{\sum_{j=1}^{n} r_j} \tag{15}$$

- Update the system according to the chosen reaction.

In this way the system can be updated, and adjusting the reactions in each time step. In Supplementary Figure 1A, we show simulations of the oscillations in NF-κB using the Gillespie algorithm, for the same initial conditions but in different volumes which results in different noise levels.

Supplementary Note 3

Transcription Factors and Hill Functions

The way that transcription factors regulate transcription is still far from well understood. However it is well established that not always only one transcription factor is needed, but rather a complex of several transcription factors act together in order to efficiently bind the polymerase and start transcription. In Supplementary Figure 1B we show a schematic figure, where three transcription factors are needed in order to bind the polymerase to the promoter region. To establish an equation for this, we consider the binding of n Transcription factors $\mathcal{T}$ to a position in on the genome E. We can write this process as:

$$\mathrm{n}\cdot\mathcal{T} + \mathrm{E} \underset{\mathrm{k_2}}{\overset{\mathrm{k_1}}{\rightleftharpoons}} \mathcal{T}_n E \tag{16}$$

From this we can define the dissociation constant as :

$$K_D^n = \frac{[\mathcal{T}]^n \cdot [E]}{[\mathcal{T}_n E]} \tag{17}$$

Now we want to consider the probability to be bound:

$$p_{bound} = \frac{[\mathcal{T}_n E]}{[E] + [\mathcal{T}_n E]} \tag{18}$$

Then we can insert the definition of the dissociation constant to obtain a Hill function on the form:

$$p_{bound} = \frac{([\mathcal{T}_n]/K_d)^n}{1 + ([\mathcal{T}_n]/K_d)^n} = \frac{[\mathcal{T}_n]^n}{K_D^n + [\mathcal{T}_n]^n} \tag{19}$$

In Supplementary Figure 1C we see the different output of the Hill function for different values of the parameters K and n. This equation is typically used to describe the production of mRNA for a system, since we describe the probability to bind to a promoter. Note that as the Hill coefficient (above n, often it is defined as h) increases, a switch like behaviour emerges for the output function and that the higher the value of K, the longer before the curve starts to rise. Many times in experiments, switch-like behaviours are found, and here the Hill equation is usually used to model this; also even though the theoretical value of n, is unknown [4]. One point to mention is here that even though a complex might consist for instance of 3 molecules, from experiments this value vary. One example is the binding of haemoglobin which should bind 4 molecules, but where experiments give a Hill coefficient of around 3.0. The constant K, should be understood as a measure for the concentration of transcription factors to occupy half of the binding sites. In general Hill functions are extremely useful to model gene regulatory functions, because they have many of the experimentally-observed required characteristics[5, 6], and suggest a natural way to implement sigmoidal signals to the mathematical description of genetic networks.

Supplementary Note 4

Protein Levels at Other Parameters

In addition to what is found in Figure 2, it is important to consider the profiles for the average number of proteins for other TNF frequencies in order to test the validity and robustness of the presented results. In Supplementary Figure 2A-F we see the resulting profiles for different values of the TNF frequency. Even though the the value of the amplitude at which chaos sets differs, the pattern show the similar characteristics, and in all cases chaotic dynamics results in an enhanced level of low affinity genes.
To test the robustness of these results even further, we tried to oscillate TNF in the presence of Langevin noise (Supplementary Figure 2G) and for these simulations, we calculated the Fourier spectrum to see the effect of the noise. The resulting protein profiles are shown in Fig. 2(G-I) in the main text, and if one considers the Fourier spectrum in Supplementary Figure 2H (below), it is clear that this is quite flat and thus this noise level almost masks the actual oscillations. The main results are still robust to this large external noise (Fig. 2G-I). In Fig 2G-I we also oscillated TNF as a Van der Pol oscillator with Langevin noise. This is described by the dynamical system:

$$\dot{x} = y + \eta(t) \tag{20}$$

$$\dot{y} = -x + \mu(1 - x^2)y + \eta(t) \tag{21}$$

With $\eta(t)$ being uncorrelated white noise. Thereby we tested different TNF waveforms and calculated the ratio of protein in the chaotic regime vs protein in the flat TNF regime (Supplementary Figure 2I). Here we find that for all the tested wave forms, chaotic up regulates the protein production with at least a factor of 2. We note that the highest effect is found when TNF is oscillated as a square pulse.
In this work the main focus have been on two genes defined by a specific value of h and K. We found (Supplementary Figure 2J) that for for increasing hill coefficients and decreasing affinity the chaotic had an increasing ratio compared to the limit cycle regime. Thus for a large interval of parameters, chaotic dynamics results in increased protein levels and if we consider genes even higher values of h and K (we cannot go much lower that Gene 1), the results are even more significant and the production for a gene with $h = 6$ and $K = 7\mu M$ has a 6 fold increase in chaos versus for flat TNF (Supplementary Figure 2H). Finally we tested how the distribution of NF-κB acutally changes in the different regimes and therefore give rise to the profiles found in Fig. 2A-C and Supplementary Figure 2A-F. Here we see that as we move into the chaotic regime, the distribution gets broader in both ends and therefore NF-κB in the chaotic regime spends more time around zero, but does also have the very large peaks that affects the low affinity genes (Supplementary Figure 2K-L).

The equation for the m_{RNA} is

$$\frac{dm}{dt} = f(t) - \delta m \tag{22}$$

$$\text{where} \tag{23}$$

$$f(t) = \frac{N(t)^h}{N(t)^h + K^h} \tag{24}$$

Because the NF-kB concentration N(t) is periodic in time with period T, therefore f(t) is also periodic with period T. To begin, let us examine the case where f(t) is composed of only a single mode with a non-zero average σ:

$$f(t) = \sigma + Ae^{i\omega t} \quad \text{with } \omega = 2\pi/T \tag{25}$$

In that case, the solution of the equation for m_{RNA} is:

$$m(t) \approx \frac{\sigma}{\delta} + \frac{A}{\delta + i\omega} e^{i\omega t} \tag{26}$$

In general of course f(t) is a Fourier sum of many modes, but since the equation for m_{RNA} is linear in m, the resultant solution is:

$$m(t) = \sum_{-\infty}^{\infty} \frac{\sigma}{\delta} + \frac{A_k}{\delta + i\omega_k} e^{i\omega_k t} \tag{27}$$

Here, the k = 0 term takes care of the σ term in the previous single-mode case. The argument can be easily extended to the protein, because the dP/dt equation is also linear in P and so each mode in m(t) will contribute additively to P(t) (just like each mode in f(t) contributes additively to m(t)). In order to understand how average expression of the gene depends on various parameters, we simply need to look at the k = 0 term, because averaged over time:

$$\langle m \rangle = \frac{A_0}{\delta} \tag{28}$$

$$\text{with} \tag{29}$$

$$A_0 = \frac{\gamma}{T} \int_0^T \frac{N(t)^h}{N(t)^h + K^h} dt = \frac{\gamma}{T} \int_0^T \frac{1}{1 + (\frac{K}{N})^h} dt \tag{30}$$

Now we consider the two cases where K is very small and very large respectively. For small K we expand the fraction:

$$A_0 \approx \frac{\gamma}{T} \int_0^T \Big(1 - \Big[\frac{K}{N(t)}\Big]^h + \mathcal{O}\Big) dt \tag{31}$$

$$= \gamma(1 - \langle (K/N)^h \rangle) \tag{32}$$

Therefore this will be dominated by the smallest values of N(t). As we see in Supplementary Figure 2K these occurs in the chaotic state, and therefore the average production in total will be smaller for chaotic than for the limit cycle case. If we instead consider the case with very large K, we make the expansion

$$A_0 = \frac{\gamma}{T} \int_0^T \frac{(\frac{N}{K})^h)}{1 + (\frac{N}{K})^h)} dt \tag{33}$$

$$\approx \frac{\gamma}{T} \int_0^T \Big(\Big[\frac{N(t)}{K}\Big]^h + \mathcal{O}\Big) dt \tag{34}$$

$$= \gamma \langle (N/K)^h \rangle \tag{35}$$

This will clearly be dominated by the largest values of N(t) and as can be seen in Supplementary Figure 2K these also occurs for the chaotic state. In this case the chaotic dynamics thus increases the average production of genes with

large K. These arguments explains the results we find in Fig. 2 in the main text from an analytical point of view in the extreme cases for the affinities of genes.

Supplementary Note 5

Overview of Complex Formation

In the following the ratios shown in Fig. 3G-I are shown. Note that each column i defined by the number og $HAGs$ in the complex.
For the presented results in Fig. 3G we used the numbers presented in the following matrix:

$R_{3G} =$

	0/n	1/n	2/n	3/n	4/n	5/n	6/n	7/n	8/n	9/n	10/n
n=2	0.619	2.18	2.67	0	0	0	0	0	0	0	0
n=3	0.606	2.08	2.18	2.78	0	0	0	0	0	0	0
n=4	0.587	1.91	2.03	2.35	3.12	0	0	0	0	0	0
n=5	0.567	1.75	1.84	2.14	2.68	3.65	0	0	0	0	0
n=6	0.546	1.54	1.68	1.94	2.39	3.13	4.42	0	0	0	0
n=7	0.526	1.41	1.54	1.76	2.15	2.76	3.78	5.59	0	0	0
n=8	0.507	1.35	1.42	1.61	1.94	2.46	3.29	4.73	7.46	0	0
n=9	0.489	1.25	1.31	1.48	1.77	2.21	2.9	4.05	6.19	10.7	0
n=10	0.472	1.16	1.22	1.37	1.62	2.0	2.59	3.53	5.2	8.61	16.6

For the presented results in Fig. 3H we used the numbers presented in the following matrix:

$R_{3H} =$

	$0/n$	$1/n$	$2/n$	$3/n$	$4/n$	$5/n$	$6/n$	$7/n$	$8/n$	$9/n$	$10/n$
$n=2$	2.81	13.8	11.7	0	0	0	0	0	0	0	0
$n=3$	2.75	13.3	9.8	12.0	0	0	0	0	0	0	0
$n=4$	2.66	12.6	8.77	9.87	13.5	0	0	0	0	0	0
$n=5$	2.57	12.1	7.99	9.28	11.6	15.7	0	0	0	0	0
$n=6$	2.48	11.1	7.28	8.39	10.3	13.6	19.1	0	0	0	0
$n=7$	2.39	10.2	6.67	7.63	9.3	12.0	16.4	24.1	0	0	0
$n=8$	2.3	9.33	6.13	6.97	8.41	10.7	14.2	20.5	32.2	0	0
$n=9$	2.22	8.67	5.67	6.41	7.66	9.57	12.6	17.6	26.8	45.9	0
$n=10$	2.14	8.04	5.26	5.92	7.02	8.67	11.2	15.3	22.5	37.3	71.7

For the presented results in Fig. 3I we used the numbers presented in the following matrix:

$R_{3I} =$

	$0/n$	$1/n$	$2/n$	$3/n$	$4/n$	$5/n$	$6/n$	$7/n$	$8/n$	$9/n$	$10/n$
$n = 2$	0.825	10.2	1.5	0	0	0	0	0	0	0	0
$n = 3$	0.734	10.9	4.58	1.85	0	0	0	0	0	0	0
$n = 4$	0.684	7.96	5.45	3.61	2.23	0	0	0	0	0	0
$n = 5$	0.647	5.86	4.65	3.92	3.32	2.7	0	0	0	0	0
$n = 6$	0.615	4.51	3.91	3.6	3.49	3.42	3.34	0	0	0	0
$n = 7$	0.587	3.64	3.33	3.21	3.3	3.52	3.83	4.25	0	0	0
$n = 8$	0.562	3.2	2.88	2.86	3.03	3.34	3.82	4.55	5.66	0	0
$n = 9$	0.539	2.75	2.52	2.55	2.75	3.1	3.62	4.41	5.73	8.02	0
$n = 10$	0.518	2.41	2.24	2.3	2.51	2.84	3.35	4.12	5.39	7.7	12.3

Supplementary Note 6

Toxicity at Other Parameters

To explore the results shown in Figure 4, we tested the distribution of proteins inside the limit cycle and chaotic dynamics. In Supplementary Figure 3A-D we show how the distribution of proteins inside the chaotic regime is much broader than for the oscillatory dynamcis. We fixed the production ratios so the average sum of the two proteins should be equal (Supplementary Figure 3C), but in the chaotic region the mixture is broader and therefore the product of the two proteins is significantly higher in the chaotic region. This is one of the explanations of why chaos increases the survival rate when several external stresses are present.

In addition to what is found in Figure 4, we consider the death rates for a series of different parameters. First, we checked the results for another TNF frequency In Supplementary Figure 3E-H we observe the situations similar to Fig. 4(I-L) but for TNF period 95 min. From these we conclude that the results are similar to the results found for TNF period 95 min, and the chaotic dynamics create increased average survival rate for the population. To test this further we tried to change other parameters and fix the hill coefficient in the death-rate expression equal to 2. In Supplementary Figure 3I we added a larger oscillation amplitude to the external stress and in Supplementary Figure 3J we added a higher stress at the peak, and find that both results are in agreement with the observations made in Fig 4K-L and Supplementary Figure 3G-H. These results might seem surprising, but there is a simple intuition of these, following the slightly simplified explanation. We defined the rate at which a cell die to be:

$$r = \mathcal{P}_0 \Big(\frac{D_1^h}{D_1^h + P_1^h} + \frac{D_2^h}{D_2^h + P_2^h} \Big) \tag{36}$$

As we defined in the text we normalized the proteins, and by adjusting their production profiles we fixed the number of total proteins. This can be seen in Supplementary Figure 3C (above), where we see that the sum of proteins has the same mean, even though the spread in the chaotic distribution is larger. Expanding the death rate (and just setting h=1), we find that:

$$r = \mathcal{P}_0 \Big(\frac{2D_1 D_2 + P_1 D_2 + P_2 D_1}{D_1 D_2 + P_1 D_2 + P_2 D_1 + P_1 P_2} \Big) \tag{37}$$

Here we note that the terms in the nominator and denominator are almost identical, but there is a product of the proteins only occurring in the denominator. Looking at the distribution of this (Supplementary Figure 3D) we see that this is significantly larger for the chaotic dynamics, since there is a mixture of represented proteins. Therefore the denominator will be larger in the chaotic regime and thus the death rate will in general be lower for the cells if they have chaotic NF-kB dynamics. For the case with the varying values of D (Fig 4K-L and Supplementary Figure 3G-J) then the intuitive explanation comes from the fact that since there is a spread of proteins then there are some outliers that will have a small rate of dying. And since these change dynamically in time, a larger fraction of the cells will have decreased death rate compared to the case for a stable limit cycle.

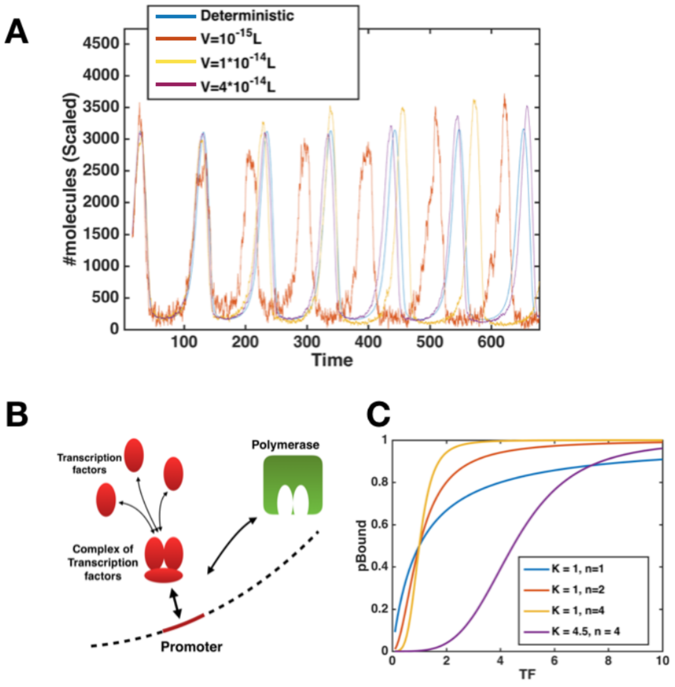

Supplementary Figure 1: Intrinsic Noise and Hill Functions. A) Simulations using Gillespie algorithm at different volumes B) Schematic figure of cooperativity in transcription factors giving rise to Hill function C) Curves of different Hill functions showing $p_{Bound}(TF) = \frac{TF^n}{TF^n + K^n}$.

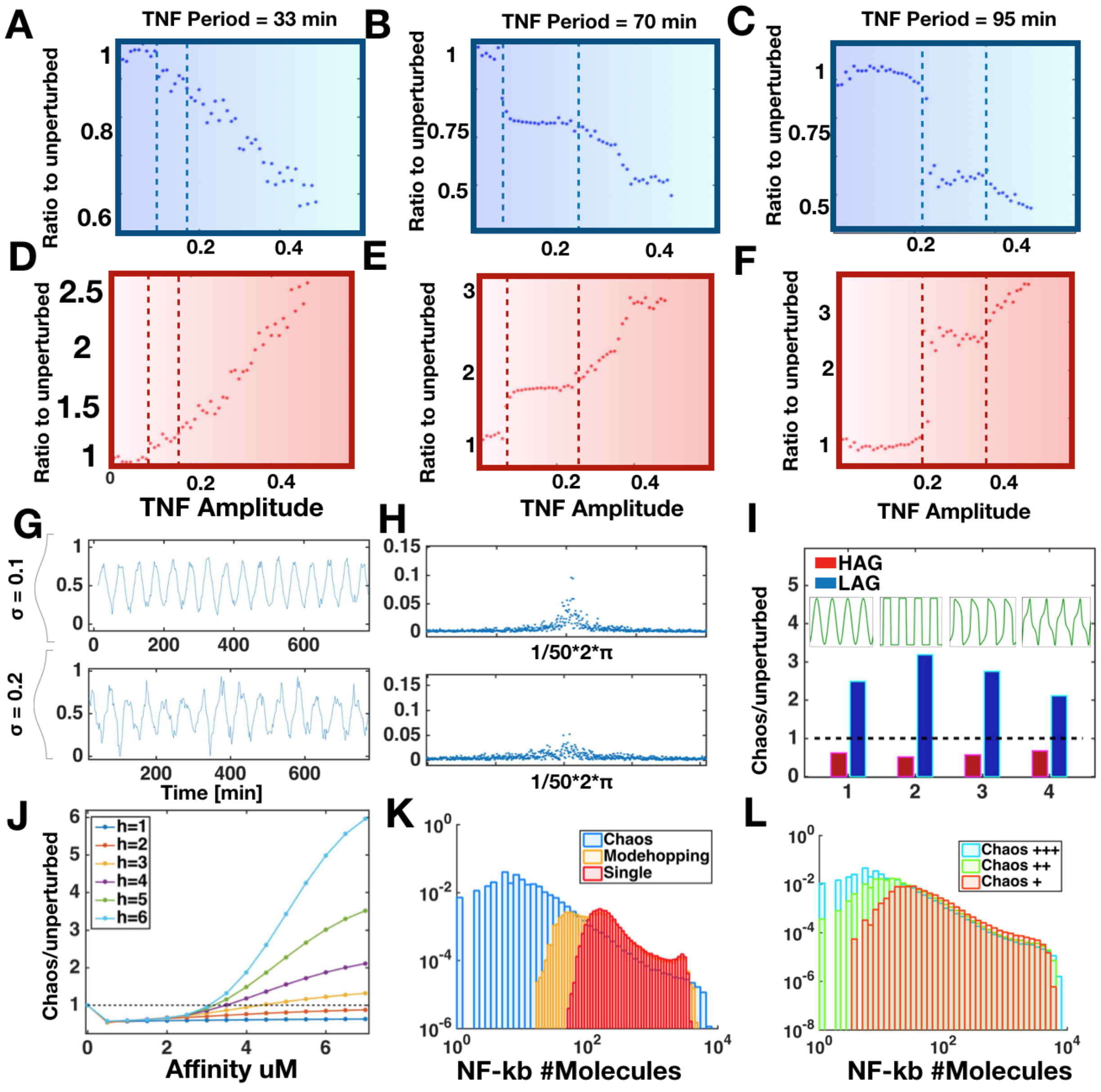

Supplementary Figure 2: Chaotic Dynamics with different parameters. A) The average production from Gene 1 in a stochastic simulation with TNF period 33 min, The results are divided in three regions, limit cycle, modehopping and chaos, reading from left to right B) The average production from Gene 1 in a stochastic simulation with TNF period 70 min C) The average production from Gene 1 in a stochastic simulation with TNF period 95 min D) The average production from Gene 2 in a stochastic simulation with TNF period 33 min E) The average production from Gene 2 in a stochastic simulation with TNF period 70 min F) The average production from Gene 2 in a stochastic simulation with TNF period 95 min G) TNF level in a Langevin simulation as a function of time, at noise level σ_1 and σ_2. H) Power spectrum of the time series shown in G. I) The ratio of Proteins at $TNF = 0.5 + 0.4sin(2\pi\omega t)$ / $TNF = 0.5$ for different wave forms. J) Protein level for $TNF = 0.5 + 0.4sin(2\pi\omega t)$ for genes defined by different levels of K and h. K) Distribution af NF-κB at different time levels inside the different regimes. L) Distribution af NF-κB at different time levels as we move deeper inside the chaotic region.

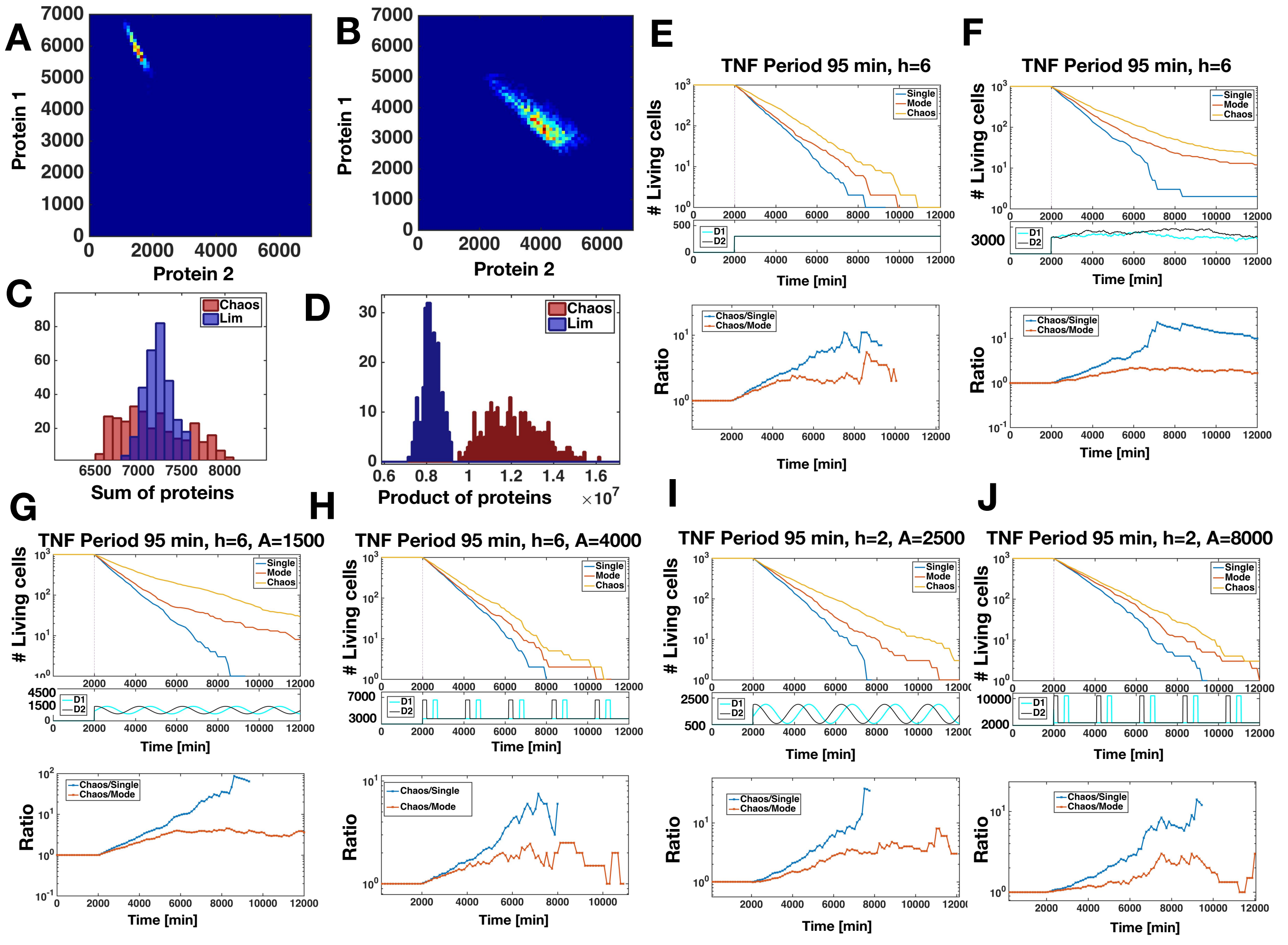

Supplementary Figure 3: Population heterogeneity and survival rate at different parameters. A) Heat map showing Protein levels during stochastic simulation when dynamics is a limit cycle. B) Heat map showing Protein levels during stochastic simulation when dynamics is chaotic. C) Sum of proteins in stochastic simulations when dynamics is in chaotic and limit cycle regime. D) Product of proteins in stochastic simulations when dynamics is in chaotic and limit cycle regime. E) Remaining cells vs time (Drug is added at T=4000 min). D1 = 3000, D2 = 3000. Below the ratio of living cells for chaos divided by limit cycle or modehopping dynamics. F) Same as A with $\dot{D}_{1+2} = \mathcal{N}(0, 100.0)$ and $D_{1+2}(0) = 3000$. The panel below shows a specific trajectory on this pattern. In general D_1 is above D_2 50% of the times and vice versa. Panels same as E. G) Same as A with $D_{1+2}(t) = 3000 + 1500 \cdot sin(\frac{t}{5000} + \Omega)$ Panels same as E. H) Same as A with $D_{1+2}(t) = 7000$ if $sin(\frac{t}{5000} + \Omega) > 0.95$ and otherwise $D_{1+2}(t) = 3000$. Here $h = 2$. Panels same as E. I) Same as A with $D_{1+2}(t) = 3000 + 2500 \cdot sin(\frac{t}{5000} + \Omega)$ Panels same as E. J) Same as A with $D_{1+2}(t) = 10000$ if $sin(\frac{t}{5000} + \Omega) > 0.95$ and otherwise $D_{1+2}(t) = 2000$ Here $h = 2$. Panels same as E.

Supplementary References

[1] Krishna, S., Jensen, M.H. & Sneppen, K.: Spiky oscillations in NF-kappaB signalling, Proc.Nat.Acad.Sci. **103**, 10840-10845 (2006).

[2] Ashall, L., Horton, C.A., Nelson, D.E., Paszek, P., Harper, C.V., Sillitoe, K., Ryan, S., Spiller, D.G., Unitt, J.F., Broomhead, D.S., Kell, D.B., Rand, D.A., See, V. & White, M.R.H.: Pulsatile stimulation determines timing and specificity of NF-B-dependent transcription. Science **324**, 242 (2009).

[3] Jensen, M.H. & Krishna, S.: Inducing phase-locking and chaos in cellular oscillators by modulating the driving stimuli, FEBS Letters **586**, 1664-1668 (2012).

[4] Phillips, R., Kondev, J., Theriot, J., Garcia, H.: *Physical biology of the cell* Garland Science (2012)

[5] Santillan, M.: On the use of the Hill functions in mathematical models of gene regulatory networks. Math. Mod. Nat. Phen. , **3(2)**, 85-97 (2008).

[6] Weiss, J. N.: The Hill equation revisited: uses and misuses., FASEB Jour., **11(11)**, 835-841 (1997).
